# A Pooled Analysis of Sex Differences in Rotaviral Enteritis Incidence Rates in Three Countries Over Different Time Periods

**DOI:** 10.1089/whr.2021.0096

**Published:** 2022-02-22

**Authors:** Victoria Peer, Naama Schwartz, Manfred S. Green

**Affiliations:** School of Public Health, University of Haifa, Haifa, Israel.

**Keywords:** incidence rate ratios, infections, rotavirus, sex differences

## Abstract

***Background:*** Sex differences in incidence rates (IRs) of infectious diseases could provide clues to the mechanisms of infection. The results of studies on sex differences in the incidence of rotaviral enteritis have been inconsistent.

***Methods:*** We carried out a pooled analysis of sex differences in IRs for rotaviral enteritis in three countries for a period of 7–22 years. Male-to-female incidence rate ratios (IRRs) were computed by age group, country, and years of reporting. A meta-analytic methodology was used to combine IRRs. Metaregression was performed to evaluate the contribution of age group, country, and years of reporting to the IRR.

***Results:*** Significantly higher IRs in males were found in the age groups 0–4, 5–9, and 10–14 years, with pooled IRRs (with 95% confidence intervals [CIs]) of 1.12 (1.09–1.14), 1.07 (1.05–1.09), and 1.13 (1.05–1.21), respectively. In adults, the sex differences were reversed with higher rates in females. The pooled male-to-female IRRs (with 95% CIs) were 0.66 (0.64–0.68), 0.78 (0.72–0.85), and 0.78 (0.72–0.84) for the age groups 15–44, 45–64, and 65+ years, respectively. Metaregression results demonstrated that age is responsible for much of the variation in IRRs.

***Conclusions:*** The higher rotaviral enteritis IRs in males at a very early age suggest that sex-related factors unrelated to exposure may play a role. The higher IRs in adult females could result, at least partly, from behavioral and occupational factors.

## Introduction

Acute diarrhea is one of the most common diseases in infants and young children all over the world and rotavirus is a common cause.^1^ Rotaviruses belong to the *Reoviridae* family and the genus *Rotavirus* is a common cause of severe diarrhea in young children. Nine rotavirus species have been described so far (A–I), and rotavirus A is the most important virus that infects humans.^2,3^ Rotaviral infection is a highly contagious disease, transmitted mainly from person to person and contaminated food.

The report of the Global Burden of Disease and extended analyses demonstrated that rotavirus infection caused 128,500 deaths and 258,173,300 episodes of diarrhea among young children in 2016.^4^ Half of the rotavirus deaths in the world occur in Nigeria, Angola, Democratic Republic of the Congo, India, and Pakistan.^5^ In 2009, to prevent life-threatening diarrhea and associated mortality, the World Health Organization (WHO) recommended the introduction of rotavirus vaccines in national immunization programs.^6^

Sex differences in the incidence of rotaviral enteritis have been reported, but the reports are based on local data sources, representing mainly infants and young children with higher incidence rates (IRs) of rotaviral enteritis in males.^7–10^ Others found no sex differences in incidence.^11,12^ Rotaviral enteritis among adults has not decreased despite effective vaccination in childhood; infections with rotavirus among adults are generally underappreciated by age and sex^13,14^ and may spread between patients, making recognition of rotaviral enteritis important for infection control.^15–17^

In general, biological sex-related regulation of the immune system can contribute to differences in incidence of infectious diseases.^18–20^

To the best of our knowledge, age-related sex differences in clinical rotaviral enteritis have not been reported over prolonged time periods. Understanding the role of sex as a variable that influences the susceptibility to clinically apparent infectious disease could contribute to our knowledge of the infection. We analyzed national data from three countries to determine the magnitude of the male-to-female incidence rate ratios (IRRs) by age group, country, and time period.

## Methods

### Search strategy

We searched for country-level data on rotavirus infections, and to guarantee comparability of data quality and collection, we restricted our search strategy to published, open-access national data disaggregated by age and sex, in which reporting of rotaviral infection is compulsory and there are similar reliable surveillance and diagnostic systems. The search was thus limited to countries in Europe, the Americas, and Australasia. The search was performed from February to May of 2018. We identified data accessible through official institutions and websites from three countries: Australia, Finland, and Germany.

Data for Australia, for years 2010–2016, were obtained from the National Notifiable Diseases Surveillance System (NNDSS)^21^; for Finland, for years 1995–2016, from the National Institute for Health and Welfare (THL)^22^; and for Germany, for years 2001–2016, from the German Federal Health Monitoring System.^23^

Information about the population size by age, sex, and year for the Australian population was obtained from the Australian Bureau of Statistics^24^; for the Finnish population from Statistics Finland's PX-Web databases^25^; and for the German population from the German Federal Health Monitoring System.^26^

### Calculation of IRs

We calculated rotaviral enteritis IRs for each country and calendar year by sex and age group together. To simplify the performance of the large amount of data in the forest plots, we consolidated calendar years together. IRs per 100,000 were calculated as the number of reported cases divided by the respective population size and multiplied by 100,000. The age groups considered were 0–4 (early childhood), 5–9 (late childhood), 10–14 (puberty), 15–44 (young adulthood), 45–64 (middle adulthood), and 65+ (senior adulthood) years.

For Finland, the data are aggregated into groups of 0–4 (early childhood), 5–9 (late childhood), 10–14 (puberty), 15–39 (young adulthood), 40–59 (middle adulthood), and 60+ (senior adulthood) years.

The male-to-female IRR for each age group, country, and time period was calculated by dividing the IR in males by that of females.

### Pooled analyses

We have previously discussed and analyzed the subject of sex differences in various infectious diseases.^27–29^ As in previous studies, we decided to pool results from the different countries using a meta-analytic methodology. The outcome variable was the male-to-female IRR. We pooled the IRRs for each age group, by country and time period. The results are presented as forest plots.

Heterogeneity between groups was tested using Cochran's *Q* statistic and *I*^2^. The statistics were used to assist in deciding on the use of the fixed or random effects model for pooling IRRs. If the *Q* test yielded a *p* < 0.1, and/or *I*^2^ ≥ 50%, the random effects model was used to pool IRRs and 95% confidence intervals (CIs). In other cases, if there still appeared to be significant heterogeneity, the random effects model was preferred.

### Sensitivity analyses

The leave-one-out sensitivity analysis was carried out to estimate the effect of each variable (country or time period) on the overall male: female IRRs. The pooled IRRs were recalculated for each age group separately.

### Metaregression analyses

To identify the main causes of variation in the IRRs, metaregression analyses were carried out. Age group, country, and group of years are the potential explanatory variables.

## Results

The summary of the male and female IRs (per 100,000 population) in different countries for each age group is presented in Table 1. In each country, the IR of rotaviral enteritis was much higher at ages 0–4 compared with all other age groups.

**Table 1.** Details of the Countries Included in the Study, by Sex and Age Group

Age, years	Country	Years	Males		Females		IRR
			***n/N***	IR	***n/N***	IR	
0–4	Australia	2010–2013	7,095/5,444,384	130.32	6,054/5,161,020	117.30	1.11
	Finland	1995–2016	11,405/3,350,820	340.36	9,235/3,209,146	287.77	1.18
	Germany	2001–2016	271,546/29,249,793	928.37	234,182/2,775,9580	843.61	1.10
5–9	Australia	2010–2013	1,425/5,250,344	27.14	1,243/4,975,038	24.98	1.09
	Finland	1995–2016	1,056/3,440,956	30.69	862/3,297,629	26.14	1.17
	Germany	2001–2016	26,189/30,760,941	85.14	23,335/29,187,252	79.95	1.06
10–14	Australia	2010–2013	372/5,028,780	7.40	241/4,773,348	5.05	1.47
	Finland	1995–2016	170/3,522,497	4.83	159/3,375,446	4.71	1.02
	Germany	2001–2016	6,573/33,455,166	19.65	5,731/31,724,889	18.06	1.09
15–44	Australia	2010–2013	1,210/34,151,695	3.54	1,732/33,680,784	5.14	0.69
	Finland	1995–2016	91/18,898,064	0.48	141/18,050,351	0.78	0.62
	Germany	2001–2016	26,314/257,895,408	10.20	38,814/247,590,330	15.68	0.65
45–64	Australia	2010–2013	797/19,780,716	4.03	1,067/20,284,996	5.26	0.77
	Finland	1995–2016	54/16,513,241	0.33	92/16,307,550	0.56	0.58
	Germany	2001–2016	19,678/181,698,132	10.83	24,444/181,849,520	13.44	0.81
65+	Australia	2010–2013	1,107/10,790,460	10.26	1,785/12,502,560	14.28	0.72
	Finland	1995–2016	158/11,159,619	1.42	225/15,066,114	1.49	0.95
	Germany	2001–2016	39,732/108,019,284	36.78	69,545/149,862,231	46.41	0.79

IR, incidence rate; IRR, incidence rate ratio; *n*, cumulative number of cases for given years; *N*, cumulative total of the population for given years.

**FIG. 1.**
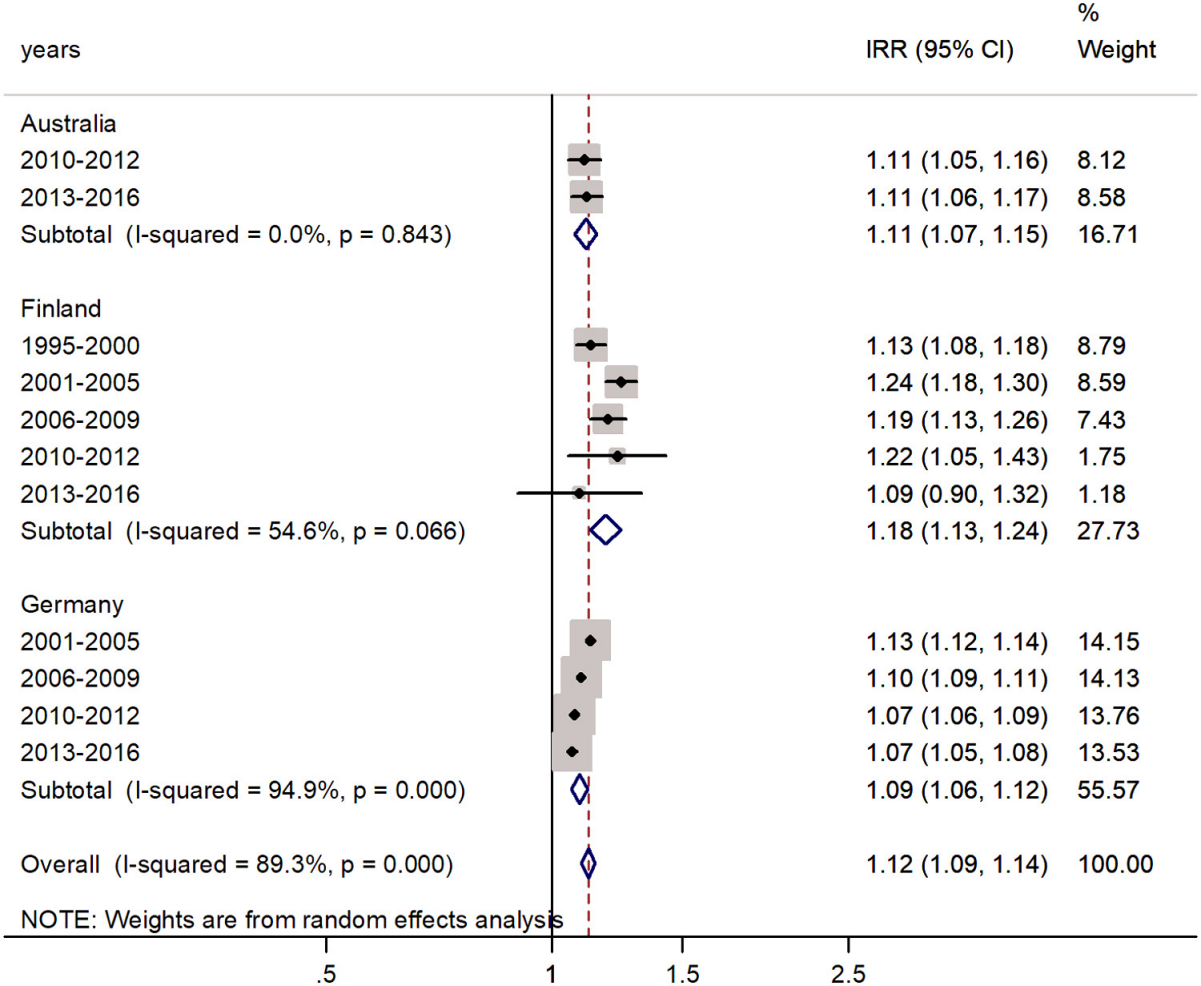
Forest plot of the male-to-female rotaviral enteritis IRRs in children aged 0–4 years for three countries by time period. IRRs, incidence rate ratios.

The forest plot for the age group 0–4 years is shown in Figure 1. The pooled male-to-female IRR was 1.12 (95% CI 1.09–1.14) with *I*^2^ = 89.3%, indicating a 12% excess in IRs in males. The highest excess was in Finland (18%) and the lowest in Germany (9%).

**FIG. 2.**
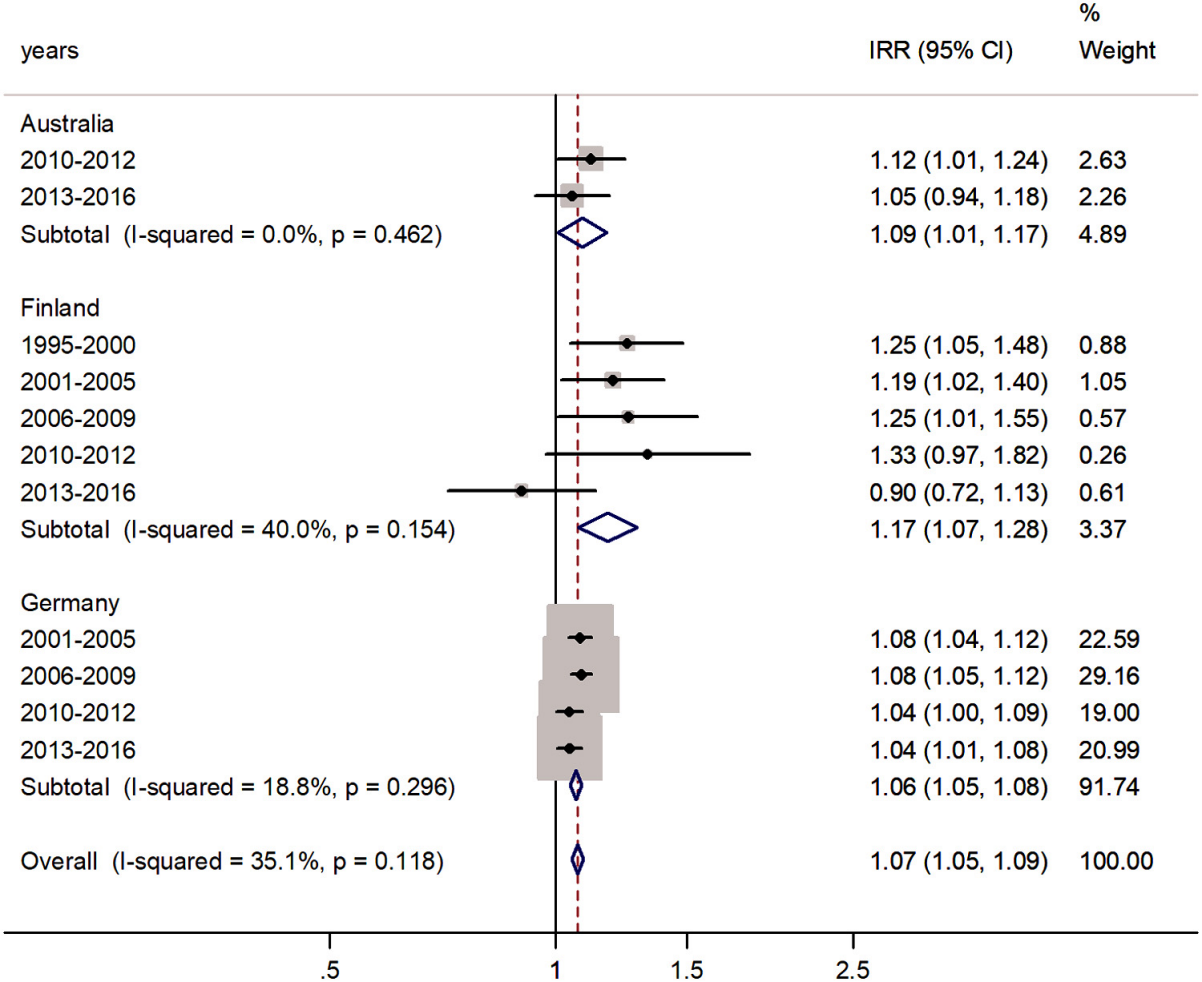
Forest plot of the male-to-female rotaviral enteritis IRRs in children aged 5–9 years for three countries by time period.

The forest plot for the age group 5–9 years is shown in Figure 2. The pooled IRR was 1.07 (95% CI 1.05–1.09) with *I*^2^ = 35.1%. The highest excess was in Finland (17%) and the lowest in Germany (6%).

**FIG. 3.**
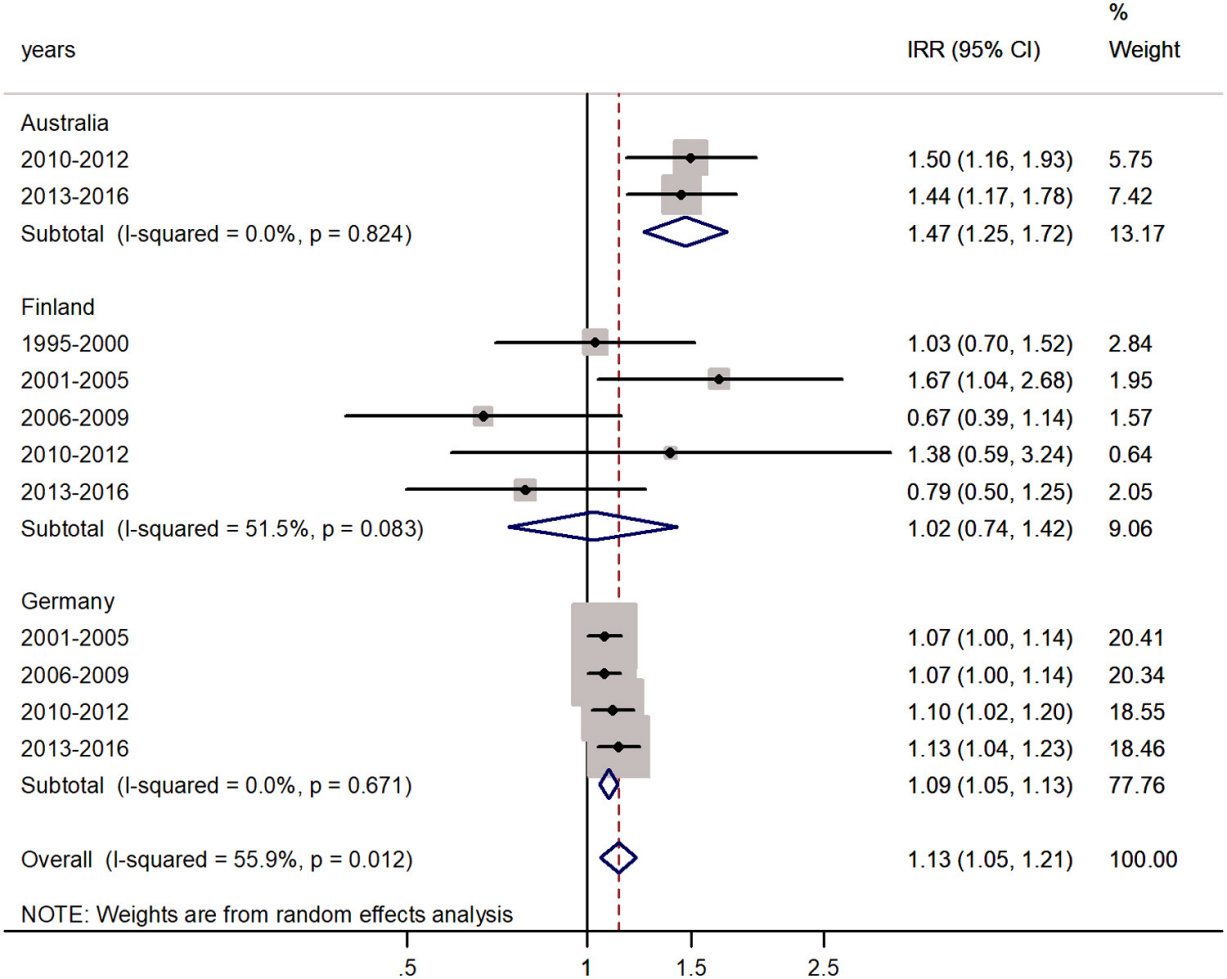
Forest plot of the male-to-female rotaviral enteritis IRRs in children aged 10–14 years for three countries by time period.

The forest plot for the age group 10–14 years is shown in Figure 3, with an overall pooled IRR of 1.13 (95% CI 1.05–1.21) with *I*^2^ = 55.9%. The highest male excess was in Australia (47%) and the lowest in Finland (2%).

**FIG. 4.**
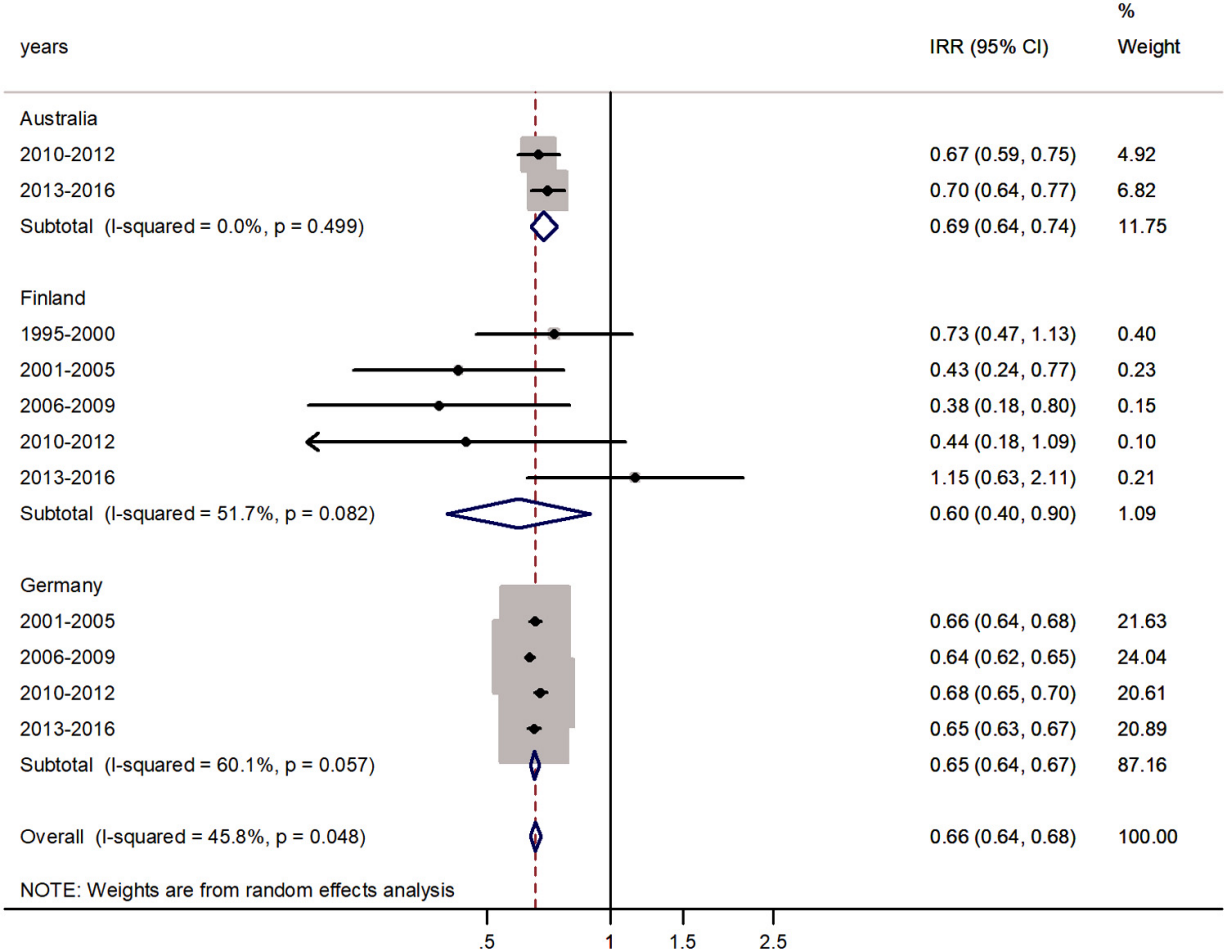
Forest plot of the male-to-female rotaviral enteritis IRRs in young adulthood (15–44 years) for three countries by time period.

The forest plots for the age group 15–44 or 15–39 years are shown in Figure 4. The IR for males declined and the male-to-female IRR was reversed, with pooled male-to-female IRR = 0.66 (95% CI 0.64–0.68) with *I*^2^ = 45.8%. A female predominance in rotaviral enteritis IRs was significant for the populations of all countries and the excess incidence in females varied from 60% in Finland to 69% in Australia.

**FIG. 5.**
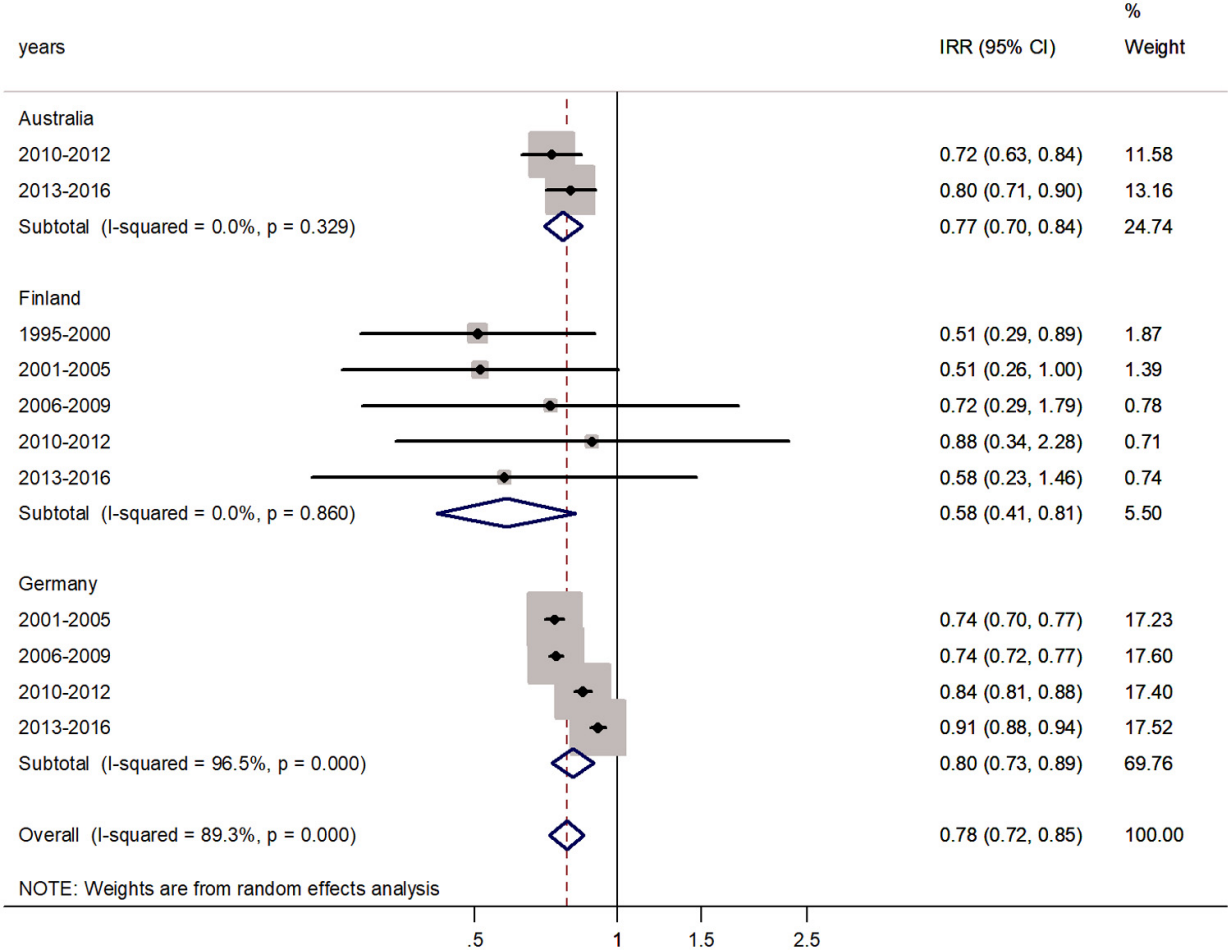
Forest plot of the male-to-female rotaviral enteritis IRRs in middle adulthood for three countries by time period.

**FIG. 6.**
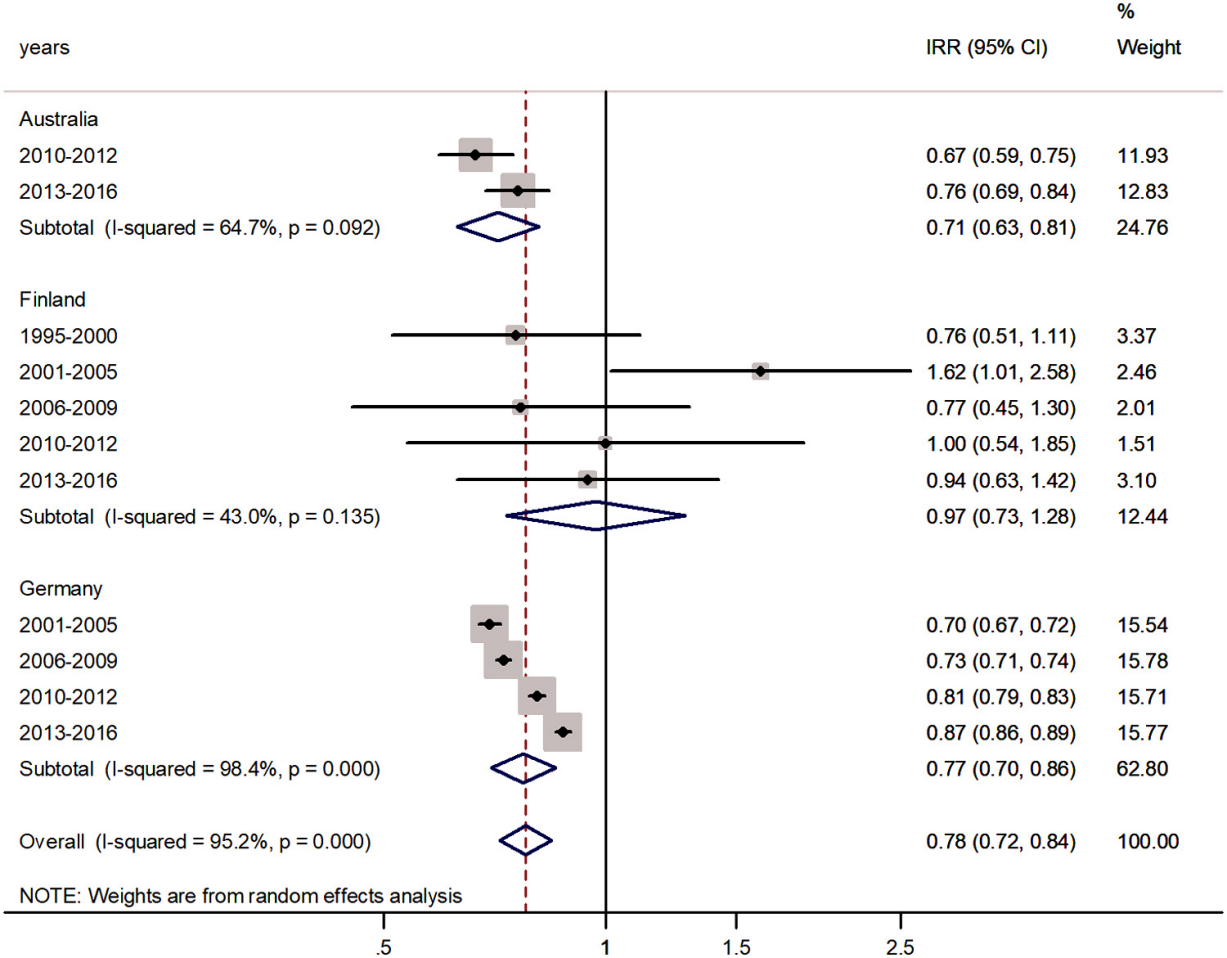
Forest plot of the male-to-female rotaviral enteritis IRRs in senior adulthood for three countries by time period.

The forest plots for middle-aged adults (45–64 or 40–59 years) and senior adults (60+ or 65+ years) are shown in Figures 5 and 6, respectively. In both age groups, significantly lower IRs were observed in males. For middle-aged adults, the male-to-female IRR was 0.78 (95% CI 0.72–0.85) with *I*^2^ = 89.3%. Excess incidence in females varied from 58% in Finland to 80% in Germany.

In senior adults, aged 65+ years, the pooled IRR was 0.78 (95% CI 0.72–0.84) with *I*^2^ = 95.2%. The highest incidence excess was in Finland (97%) and the lowest in Australia (71%).

### Sensitivity analysis

To evaluate the effect of each of the countries and time periods on the pooled IRRs in each age group, we performed sensitivity analysis and recomputed the pooled IRRs (represented in Tables 2 and 3, respectively). After omission of one country at a time, the pooled rotaviral enteritis male-to-female IRRs remained relatively unchanged except in puberty, when omission of data from Finland or Germany impacted the pooled IRRs (IRR = 1.25, 95% CI 0.93–1.67, after omission of data from Finland and IRR = 1.23, 0.87–1.75, after omission of data from Germany, respectively).

**Table 2.** Sensitivity Analysis, by Age Group and Country

Age group Country removed	Early childhood IRR (CI)	Late childhood IRR (CI)	Puberty IRR (CI)	Young adulthood IRR (CI)	Middle adulthood IRR (CI)	Senior adulthood IRR (CI)
Australia	1.14 (1.06–1.22)	1.07 (1.05–1.09)	1.09 (1.05–1.12)	0.65 (0.64–0.66)	0.71 (0.52–0.97)	0.84 (0.71–0.99)
Finland	1.1 (1.09–1.11)	1.07 (1.05–1.08)	1.25 (0.93–1.67)	0.66 (0.63–0.7)	0.8 (0.78–0.83)	0.76 (0.69–0.84)
Germany	1.15 (1.08–1.22)	1.12 (1.06–1.19)	1.23 (0.87–1.75)	0.68 (0.64–0.73)	0.7 (0.54–0.9)	0.81 (0.62–1.06)

CI, confidence interval.

**Table 3.** Sensitivity Analysis, by Time Period and Age Group

Age group Years removed	Early childhood IRR (CI)	Late childhood IRR (CI)	Puberty IRR (CI)	Young adulthood IRR (CI)	Middle adulthood IRR (CI)	Senior adulthood IRR (CI)
1995–2000	1.09 (1.07–1.12)	1.07 (1.05–1.09)	1.1 (1.06–1.15)	0.66 (0.64–0.67)	0.8 (0.73–0.88)	0.76 (0.7–0.83)
2001–2005	1.09 (1.07–1.11)	1.07 (1.05–1.09)	1.11 (1.06–1.16)	0.66 (0.63–0.68)	0.81 (0.72–0.91)	0.78 (0.72–0.86)
2006–2009	1.1 (1.06–1.14)	1.06 (1.04–1.08)	1.12 (1.07–1.16)	0.66 (0.65–0.68)	0.81 (0.72–0.9)	0.78 (0.7–0.86)
2010–2012	1.1 (1.08–1.13)	1.07 (1.05–1.09)	1.09 (1.05–1.14)	0.65 (0.64–0.66)	0.77 (0.68–0.88)	0.76 (0.67–0.85)
2013–2016	1.11 (1.08–1.13)	1.08 (1.06–1.1)	1.09 (1.05–1.13)	0.65 (0.63–0.68)	0.76 (0.7–0.83)	0.74 (0.69–0.79)

Similar results were received after performing the sensitivity analysis for evaluation of the impact of group of years (Table 3) after the omission of one time period at a time. This confirms that the results of this meta-analysis are consistent.

### Metaregression analysis

Metaregression results revealed that age group (*p* < 0.001) accounted for the main source of heterogeneity. Across age groups, there was no significant difference between early and late childhood and between early childhood and puberty (*p* > 0.05). The heterogeneity for Germany and Finland resulted from differences in the sex ratios between the time periods. We assume that heterogeneity over time is probably due to random variation.

## Discussion

In the current study, we used a pooled analysis with meta-analytic statistical methodology to examine the male-to-female IRRs by age group in three countries over a period of 7–22 years. The highest IRs at all ages were observed in Germany. This may be, at least partly, explained by low overall vaccination coverage compared with other countries with vaccination policies.^30^

Our study showed that IRs were 12%, 7%, and 13% higher in males in age groups of 0–4, 5–9, and 10–14 years, respectively.

After puberty, the trend was reversed. The IRs were 66%, 78%, and 78% higher in females in the age groups 15–44, 45–64, and 65+ years, respectively. To the best of our knowledge, this is the first report on a detailed estimation of sex differences in rotaviral enteritis for specific age groups, from a number of countries in different geographic areas, and over long time periods. Other previous studies varied in methodology and referred to a specific age or group of years.^7–12^

This study has several strengths. We used population denominators (population size by age and sex in a specific calendar year) to evaluate IRs. The numbers of cases were based on national data from three countries with large populations and numbers of cases for an extended period of years. The countries are likely to be largely similar to other Western countries in socioeconomic level, health systems, and lifestyle. This study presents data from countries where there is no cultural preference toward male children when making the decision for hospitalization or seeking health care.

There are also limitations. The methods of rotavirus laboratory diagnosis, clinical anamnesis, or differences in reporting policies may not be identical across countries and through years, although they are unlikely to differ between males and females. Underreporting and criteria of rotavirus laboratory diagnosis could be sources of information bias, but should not differ between sexes.

Since we could not access data from African and Asian countries, it may not be possible to generalize our findings to countries or cultures with different socioeconomic statuses and lifestyles. A possible source of selection bias could be selective sex-based immunization in children. There is no evidence to suggest that this may be the case in the countries studied. In the older population, there may sex differences in utilization of health services.^31^ We assume that even if there is geographic variation in the rotavirus subgroups, it should not be related to sex differences.

An excess in rotaviral infections has been observed in other studies. In a study in Nigeria on 7–12-month-old children, rotaviral enteritis was more common in males.^7^ This is in agreement with findings regarding increased susceptibility of boys to rotaviral enteritis.^32^ In a study on 827 children at age 0 to 144 months in Shiraz, 62% of 347 admitted patients with rotaviral enteritis were males.^32^

In contrast, in a study of hospitalized children in Beijing during the period 2010–2014, 692 cases of 3,147 were positive for rotavirus, and there was no association between the sex of participants and the detection rates of rotavirus.^33^ A study of 5–14-year-old children in Germany revealed higher, but insignificant, sex differences.^8^

The incidence of the disease in adults, especially in females, has not been studied enough.^15–17^

Many studies document differences in the clinical manifestations of viral diseases and in the immune response between sexes.^19,20^ The exact biological mechanisms involved remain poorly understood. Various factors may explain the sex differences in clinical rotaviral enteritis. They include behavioral, hormonal, and genetic factors. In addition, differences in the microbiota as well as the varied efficacy of vaccines in childhood may contribute to sexual dimorphism. Rotavirus is usually transmitted by the fecal–oral route through person to person and contaminated food or water. It is highly contagious and easily transmissible.

Serum titers of rotavirus-specific antibodies were considered to be predictors for protection against the rotavirus infection.^34^ The overall increase in both male and female IRs in the older adult group may be explained by decreased activity of the immune system as a result of physiological and biological changes^18^ and may be linked to aging, waning immunity, and crowding. The change in distribution of circulating rotavirus viruses also could be a potential cause for an increase in incidence of infection in the age group of adults and elderly.^15^

The other possible explanation may be the assumption that rotavirus vaccines have no herd effect in adults outside the epidemic season. In adults, exposure differences could be important factors for higher incidence of infection in females. At older age, it is likely that females are more exposed to rotavirus during the care of infants and young children and possibly in occupational and health care settings.^35^

This may explain, in part, the excess in IRs in adult females.

Sex-related factors could affect the immune response to rotaviral infection. The rotavirus entry into the cell stimulates the innate immune response by recognition of pathogen-associated molecular patterns by host Toll-like receptors and type I interferon (IFN) expression, which are mediated by macrophages and cytokines.^36^ Estrogen promotes innate immune system pathways, including production of type I IFN,^37^ possibly inhibiting virus entry, which may be related to the ability of estradiol to increase expression of IFN-induced antiviral genes.^38^

As we mentioned above, genetic factors play a role in regulating the immune modulation of immune-related genes; Toll-like receptors such as TLR7 and TLR8 are important for rotavirus recognition and are encoded on the X chromosome, leading to gene dosage effects that may be relevant for immune response.^39^

We assume that in young children, sex differences in the incidence of clinical disease are not due to differences in exposure to the virus. In schoolchildren, sex-related differences were observed in food consumption.^40^ Differences in rotaviral enteritis IRs are unrelated to exposure to the virus and could reflect behaviors that influence exposure to rotavirus. In infants and young children, sex differences in exposure or medical service utilization are unlikely to dominate.

In very young children, hormonal and genetic component differences may contribute to sexual dimorphism. Minipuberty has been suggested as a critical window of programming with lifelong implications. In infant boys, the postnatal rise in luteinizing hormone and follicle-stimulating hormone is associated with increase in testosterone. In girls, the endogenous production of estrogen increases after birth and estradiol levels fluctuate, decreasing gradually toward the second year of life.^41^

Thus, the excess in rotaviral infections in childhood in males may be related to an interaction between hormonal and chromosomal differences.

Sex differences in the gut microbiota have been observed and may play a role in the sex dimorphism in rotavirus infection incidence.^42^ The interactions between the microbiota, hormones, pathogens, and intestinal immune factors could be reciprocal with each impacting the other.^18,43^

In assessing sex differences, the possible impact of rotavirus vaccines should be considered. Oral vaccines have been shown to be safe and effective in young children. To the best of our knowledge, studies have not demonstrated different responses in boys and girls.^44,45^ The vaccines have been available in Australia, Canada, and Finland since 2006, 2008–2010, and 2009, respectively, with no evidence on differences in vaccination rates by sex.^45–47^ In our study, sex differences in rotaviral enteritis IRs did not change during the prevaccination versus postvaccination periods.

## Conclusions

In conclusion, sex-specific factors may explain, at least in part, the consistent higher incidence of rotaviral enteritis in young males. In adults, the female predominance may be largely due to exposure to infected children. Future studies should explore pathways and immune responses that differ between the sexes. The female preponderance observed in rotaviral enteritis in adulthood provides new insights into the possible impact of gender-specific exposure on the incidence of the disease.

## Ethics Approval and Consent to Participate

National, open-access, aggregated anonymous data were used and there was no need for ethics committee approval.

## Availability of Data and Materials

All data generated or analyzed during this study are included in this article (Table 1).

## Abbreviations Used

CI: confidence interval
FSH: follicle-stimulating hormone
IFN: interferon
IR: incidence rate
IRRs: incidence rate ratios
LH: luteinizing hormone
NNDSS: National Notifiable Diseases Surveillance System
THL: National Institute for Health and Welfare
TLR: Toll-like receptor
WHO: World Health Organization
